# A multidimensional approach to the self in non-human animals through the Pattern Theory of Self

**DOI:** 10.3389/fpsyg.2025.1561420

**Published:** 2025-04-09

**Authors:** Matteo Laurenzi, Antonino Raffone, Shaun Gallagher, Salvatore G. Chiarella

**Affiliations:** ^1^Department of Psychology, Sapienza University of Rome, Rome, Italy; ^2^Department of Philosophy, University of Memphis, Memphis, TN, United States; ^3^School of Liberal Arts (SOLA), University of Wollongong, Wollongong, NSW, Australia; ^4^International School for Advanced Studies (SISSA), Trieste, Italy; ^5^Department of International Humanities and Social Sciences, UNINT, Rome, Italy

**Keywords:** self, non-human animals, pattern theory of self, multidimensional framework, non-hierarchical approach, gradedness

## Abstract

In the last decades, research on animal consciousness has advanced significantly, fueled by interdisciplinary contributions. However, a critical dimension of animal experience remains underexplored: the self. While traditionally linked to human studies, research focused on the self in animals has often been framed dichotomously, distinguishing low-level, bodily, and affective aspects from high-level, cognitive, and conceptual dimensions. Emerging evidence suggests a broader spectrum of self-related features across species, yet current theoretical approaches often reduce the self to a derivative aspect of consciousness or prioritize narrow high-level dimensions, such as self-recognition or metacognition. To address this gap, we propose an integrated framework grounded in the Pattern Theory of Self (PTS). PTS conceptualizes the self as a dynamic, multidimensional construct arising from a matrix of dimensions, ranging from bodily and affective to intersubjective and normative aspects. We propose adopting this multidimensional perspective for the study of the self in animals, by emphasizing the graded nature of the self within each dimension and the non-hierarchical organization across dimensions. In this sense, PTS may accommodate both inter- and intra-species variability, enabling researchers to investigate the self across diverse organisms without relying on anthropocentric biases. We propose that, by integrating this framework with insights from comparative psychology, neuroscience, and ethology, the application of PTS to animals can show how the self emerges in varying degrees and forms, shaped by ecological niches and adaptive demands.

## 1 Introduction

Over the past decade, the scientific community has witnessed a growing interest in the study of consciousness in non-human animals. Landmark declarations, such as the Cambridge Declaration on Consciousness (Low et al., 2012) and the more recent New York Declaration (Andrews et al., 2024), have catalyzed interdisciplinary engagement across neuroscience, comparative psychology, animal welfare science, philosophy, and evolutionary biology. These efforts have been driven by the growing recognition of the importance of understanding animal consciousness not only in terms of observable behavior but also in its intrinsic, subjective experience (Baars, 2005; Birch et al., 2022; Browning, 2022; Browning and Veit, 2022; Lecorps et al., 2021; Veit, 2022), deserving the same rigor and depth traditionally reserved for the study of consciousness in human beings (Crump and Birch, 2022; Griffin and Speck, 2004).

Despite these advances in the field of animal consciousness, a significant dimension of animal experience appears still underexplored, namely, the self. Recent theories addressing consciousness in animals often incorporate facets of the self as components of their broader models of consciousness (e.g., Birch et al., 2020; Dung and Newen, 2023; Lage et al., 2022). However, while consciousness and self are closely interrelated, the two are not synonymous. These approaches, while illuminating, tend to treat the self as secondary to or derivative of consciousness (Frewen et al., 2020; Morin, 2006; Zahavi, 2000) and risk overlooking the unique and independent dimensions of the self, which warrant focused examination (see Alcaro et al., 2017; Bekoff and Sherman, 2004; DeGrazia, 2009; Dobos and Pongrácz, 2024; Lei, 2023; Northoff and Panksepp, 2008; Thomas, 2016; Woodford, 2023).

Traditionally, the self both in humans and non-human animals has been conceptualized through a dichotomous framework, distinguishing between low-level, minimal aspects, associated with bodily, affective, and phenomenological aspects, and high-level, cognitive and conceptual dimensions, such as self-recognition or the understanding of the mental states of others (Gallagher, 2000; James, 1892; Tagini and Raffone, 2010). In line with this approach, studies have reported different aspects of the self in animals across different species. Classical studies often concentrate on specific aspects of the self, mostly related to self-recognition, theory of mind and metacognition (e.g., Gallup, 1982; Gallup and Anderson, 2020; Horschler et al., 2020; Lage et al., 2022). However, crucial dimensions are often excluded from the broader picture, ranging from subjective, phenomenological and bodily experiences to normative or extended factors. Only recently studies have started to investigate aspects related to bodily self-awareness and deeper extended dynamics across different species (e.g., Dobos and Pongrácz, 2024; Lei, 2023; Lenkei et al., 2020; Pongrácz et al., 2023; Krieger et al., 2020; Jékely et al., 2021). Given the growing interest in cognitive functions of non-human animals, we emphasize the need for a theoretical framework capable of addressing a broader spectrum of self-related features.

## 2 A multidimensional approach to the study of self in animals

We propose an integrated approach to studying the self in non-human animals, grounded in the notion of the self as a multidimensional construct (e.g., Feng et al., 2018; Zahavi, 2000). The candidate framework we propose is the Pattern Theory of Self (Gallagher, 2013; Gallagher and Daly, 2018), which characterizes the self as a pattern of interrelated factors that are dynamically related in varying degrees. A key strength of the Pattern Theory of Self (PTS) lies in its conceptualization of the self as a multidimensional construct that arises from the interaction of multiple contributing aspects, none of which is individually necessary or sufficient. We argue that this multidimensional, dynamic, and holistic perspective may account for both inter- and intra-species variability while minimizing the risk of anthropocentric bias when defining the self in animals (Brebner et al., 2024; Milton, 2020; Servais, 2018). Oversimplification inherent in one- or two-dimensional models often fails to account for the variability and adaptability of self-patterns across species, contexts, and developmental stages. For example, single-axis frameworks may overlook the interaction between bodily awareness, cognitive capacities, and intersubjective interactions that collectively shape an individual’s sense of self (Davey and Harrison, 2022; Quigley et al., 2021).

A similar multidimensional perspective has been recently proposed for the study of consciousness in non-human animals by Birch et al. (2020), in which the authors proposed five independent dimensions: “Perceptual richness,” which refers to the level of sensory detail with which an animal experiences the world; “Evaluative richness,” which captures the depth and diversity of an animal’s affective states; “Integration at a time (unity),” which describes how cohesive an animal’s conscious experience is at a given moment; “Integration across time (temporality),” which refers to the capacity to link experiences across time, including episodic memory and planning; and “Self-consciousness (selfhood),” which relates to an animal’s awareness of itself as distinct from the environment. In essence, this model provides a taxonomy for studying consciousness in non-human animals by recognizing that species may exhibit different consciousness profiles.

Similarly, the advantage of a multidimensional and dynamic framework becomes particularly salient when addressing long-standing issues in the study of self-awareness, such as whether it is graded- or state-based (Cohen et al., 2023; Sy et al., 2021). Theories that emphasize a graded approach suggest that self-awareness exists on a continuum, varying in complexity and expression across different organisms. Conversely, state-based theories posit that self-awareness is an all-or-nothing phenomenon, emerging only under specific conditions (Overgaard and Sandberg, 2021). Both perspectives offer valuable insights, yet their reliance on narrow dimensions has limited their explanatory power. A multidimensional framework, such as the one offered by the PTS, can reconcile these perspectives by demonstrating how different dimensions of the self may manifest in varying degrees or states depending on the organism and context. We argue for the importance of expanding the current focus on animal consciousness to include a deeper exploration of the self. By doing so, we aim not only to enhance our understanding of animal cognition and behavior but to further inform broader philosophical and ethical considerations regarding the lives and welfare of non-human animals (Homberg et al., 2024).

### 2.1 The Pattern Theory of Self

PTS offers a dynamic, multidimensional framework for the study of self. Unlike traditional theories, PTS conceptualizes the self as an emergent pattern of interrelated dimensions that are dynamically related but not hierarchically arranged (Gallagher, 2013, 2024; Gallagher and Daly, 2018). In the following sections, we outline the key features of the PTS and the model advocated by Birch et al. (2020). By comparing these frameworks, we aim to elucidate the benefits of a multidimensional approach to the study of the self in animals. The elements proposed by the PTS are the following:

*Bodily processes* which include core biological, ecological and interoceptive factors, allowing the system to distinguish between itself and what is not itself (e.g., other entities in the environment): a distinction crucial for survival behaviors. This element parallels, in part, the construct of “Perceptual richness” proposed by Birch et al. (2020), conceived as the level of detail and complexity with which an animal perceives its environment through sensory modalities. Studies emphasize that even the simplest nervous system supports a basic form of embodied perception, allowing organisms to establish a self vs. non-self distinction and to respond adaptively to their surroundings (Schmitt and Schoen, 2022).

*Minimal experiential processes* include subjective experiences characterized by egocentric perspective, which arise through sensorimotor integration of external and internal sensory inputs (including proprioception) that allow for a sense of self at a time. These experiential aspects include a pre-reflective sense of ownership and sense of agency (see Blanke and Metzinger, 2009; Gallagher, 2000; Levin, 2022). This experiential aspect of the self-pattern parallels the construct of “Integration at a time (unity)” proposed by Birch et al. (2020), conceived as the ability of an animal to unify sensory information into a cohesive, singular conscious perspective at any given moment. Several studies reported that minimal experiential processes are widespread, supporting the idea of a shared mechanisms for bodily ownership and agency across the animal kingdom (DeGrazia, 2009; Bradshaw and Holzapfel, 2010; Bennett and Hill, 2014).

*Affective aspects* include affect, valence, emotion, and temperament ranging from bodily sensations to broader emotional patterns. This dimension is parallel to the “Evaluative richness” proposed by Birch et al. (2020), conceived as the diversity and depth of an animal’s emotional experiences, including positive and negative valence, which influence affect-based decision-making and behavioral responses. Emotional states indeed cover a central role in both frameworks in influencing behavior and decision-making.

*Intersubjective aspects* subtend the capacity for attuning relations with other beings, which develops into social self-awareness, understanding of others’ intentions, and self-for-others, facilitating cooperation driven by empathy and altruistic behaviors. These elements do not find specific parallel in Birch’s model. However, it is worth noting that many intersubjective processes may be found in the animal kingdom. Elephants display profound mourning behaviors when a herd member dies, demonstrating deep emotional connections when their companions die (Douglas-Hamilton et al., 2006). Similarly, rats exhibit empathy-driven behavior, such as freeing trapped cage-mates, suggesting the presence of altruistic and empathetic capacities (Bartel and Orrock, 2022).

*Psychological and cognitive elements* emphasize reflective self-awareness, self-recognition, and conceptual understanding but also psychological continuity, with memory playing a critical role in personal identity. This aspect is parallel to the “Self-consciousness (selfhood)” in Birch model, conceived as the awareness of itself, ranging from bodily awareness to more abstract self aspects.

Mirror self-recognition (MSR) has been traditionally investigated as an indicator of self-awareness, primarily in great apes (Gallup, 1977; Gallup and Anderson, 2020). Further research on self-recognition has expanded to include a broader range of species, like dolphins (Reiss and Marino, 2001), elephants (Plotnik et al., 2006), dogs (Horowitz, 2017), and horses (Baragli et al., 2017), as well as magpies (Prior et al., 2008), manta rays (Ari and D’Agostino, 2016), cichlids (Thünken et al., 2009), squids (Ikeda and Matsumoto, 2007), and cleaner wrasse (Kohda et al., 2022). However, these studies indicated the need for a more nuanced interpretation beyond anthropocentric frameworks (e.g., Gallup and Anderson, 2018; Hotta et al., 2018). Furthermore, cognitive abilities of non-human primates are widely investigated, and for instance, orangutans and capuchin monkeys demonstrate an impressive capacity for abstraction, as seen in their ability to plan and use tools hours in advance, strengthening the argument for psychological continuity in these species (e.g., Metcalfe, 2013; Ottoni and Izar, 2008; van Schaik et al., 1996; van Schaik et al., 2013).

*Reflective capacities* involve the ability to regulate one’s actions and desires. Such capacities for reflection are linked to autonomy and involve second-order volitions (Frankfurt, 1988). These capacities have no parallel in the model do Birch’s model. However, great apes exhibit behaviors suggesting intentional deception, indicating a second-order volition and an understanding of others’ mental states (Byrne and Whiten, 1992). Similarly, New Caledonian crows demonstrate reflective problem-solving when modifying tools to extract food (Hunt, 1996).

*Narrative capacities* refer to aspects of self that are constructed through stories, allowing the creation of a sense of self across time. This aspect is in parallel to the “Integration across time (temporality)” in Birch’s model, conceived as the capacity of an animal to connect experiences over time, including memory recall and future planning, forming a continuous stream of conscious experiences. Some animals exhibit behaviors suggesting temporal continuity or a form of proto-narrative. Elephants, for example, revisit sites tied to significant life events, such as areas where herd members have roamed, reflecting a rudimentary narrative sense (McComb et al., 2006). Similarly, scrub jays illustrate narrative-like capacities by demonstrating awareness of future needs during food caching (Raby et al., 2007).

*Ecological (extended and situated) elements* are expressed through material engagement with belongings, tools and technologies, which shape possibilities for action and self-agency. These elements have no parallel in Birch’s model. Yet, hermit crabs, for instance, rely on external objects (shells) for protection (Briffa and Twyman, 2011), while similarly, chimpanzees that use tools to hunt or forage demonstrate the integration of external resources into their agency (Sanz and Morgan, 2013).

*Normative factors* are derived from living in family and culture, which, in the human, influence social self-understanding (e.g., who I am in terms of what role I play), individual action and societal interactions. These factors have no parallel in the model proposed by Birch. Meerkat societies, for example, engage in cooperative breeding, where individuals assist in rearing offspring that are not their own, reflecting established social norms within their groups (Clutton-Brock et al., 2001). Marine mammals, like orcas, demonstrate culturally transmitted hunting techniques, such as intentionally beaching to catch seals, which vary between pods and reflect learned behaviors within distinct social groups (Rendell and Whitehead, 2001).

## 3 Characteristics of the Pattern Theory of Self for the study of self in animals

To propose the adoption of a multidimensional perspective in the study of non-human animals, we discuss three key features of PTS. We aim to show how the PTS manages to account for the diverse ways in which environmental pressures, social structures, and cognitive capacities influence the development and expression of the self.

### 3.1 Gradedness

Gradedness is central to PTS: the dimensions constituting the self are not uniformly developed or expressed (Gallagher, 2013; Gallagher and Daly, 2018; Gallagher et al., 2024). Instead, they emerge in varying degrees of strength or prominence and can have different weights within the self-pattern. For instance, an individual, influenced by cultural and societal norms, may have a more developed normative sense, whereas another may exhibit more pronounced narrative talents, rich in personal storytelling and memory coherence. Similarly, animal species may exhibit stronger development in certain dimensions over others, reflecting their unique ecological niches and adaptive needs. This characteristic may account for intra- and inter-species variability. Within a single species, gradedness of self-dimensions may be evident in the diversity of experiences and capacities. Consider, for example, intersubjective processes: some individuals may possess exceptional social attunement compared to others of the same species, enabling deep empathic connections and successful navigation of complex social environments, while others may demonstrate stronger reflective capacities compared to others of the same species, allowing superior planning abilities and even moral deliberation. Conversely, gradedness may be also observable across different species, as a reflection of evolutionary adaptation of self-dimensions to specific environmental and survival demands. For instance, intersubjective processes in chimpanzees are highly developed, as they recognize themselves in mirrors, form complex social alliances and engage in cooperative behaviors (Hecht et al., 2017; Krachun et al., 2019). By contrast, species such as cephalopods (e.g., octopuses) may display minimal intersubjective self-dimensions but exhibit remarkably situated/extended selves, evident in their use of tools and environmental manipulation (Finn et al., 2009; Kuba et al., 2006; Mather, 2022).

### 3.2 Non-hierarchical multidimensionality

The PTS posits a non-hierarchical multidimensionality, where no one of the dimensions is essential or required to constitute the self-pattern. While humans typically embody all dimensions to varying degrees, different species may exhibit only some dimensions, operating in combination without any being foundational. While this is true for differences across species, within each species we argue for a lower or minimal variability. Within humans, for example, this is evident in individuals with neurodevelopmental conditions (i.e., patients with autism may exhibit diminished intersubjective capacities while retaining strong narrative or reflective dimensions; Du Bois et al., 2014; Delafield-Butt et al., 2020). Across species PTS’s non-hierarchical framework accommodates a variety of ways in which the self manifests in the animal kingdom. For example, ants, as eusocial insects, may exhibit affective dimensions, such as pheromone-driven states (Jackson and Morgan, 1993; von Thienen et al., 2014), but likely lack reflective or narrative dimensions entirely. On the other hand, elephants may possess affective and intersubjective dimensions (Byrne et al., 2008; Hope et al., 2025) alongside something like narrative capacities, evidenced by their long-term memory and recognition of death (Bradshaw, 2009; Goldenberg and Wittemyer, 2020). Therefore, while some animals express a broad array of dimensions, others may operate effectively with a minimal set of aspects.

### 3.3 Flexible adaptivity

Flexible adaptivity emphasizes the capacity of the self-pattern to adapt to environmental stimuli and internal motivations. This adaptability enables individuals, whether human or non-human, to recalibrate and express different dimensions of self in response to new contexts or challenges. Importantly, this flexibility applies across all dimensions of the PTS, from the bodily-oriented (e.g., minimal experiential processes) to the metacognitive ones (e.g., narrative processes). Dimensions can shift in prominence or shape based on external influences or internal drives. Within humans, the adaptive flexibility of self-dimensions is evident across life stages and personal experiences (Fadjukoff et al., 2016; Waterman, 1982). For instance, during adolescence, the narrative self may undergo significant evolution, as individuals recalibrate their understanding of identity in response to shifting social roles and internal reflections (Branje, 2022; Pasupathi and Hoyt, 2009; Van Doeselaar et al., 2020). Similarly, practices such as mindfulness or meditation can reshape one’s minimal experiential processes, refining bodily awareness and altering sensory-motor engagement (Chiarella et al., 2024; Kerr et al., 2013; Naranjo and Schmidt, 2012). This flexibility also extends to normative or reflective capacities, where exposure to diverse cultural or ethical frameworks prompts a person to reassess values and goals (Crowne, 2013; Vylobkova and Heintz, 2023).

The adaptive flexibility of self-dimensions is equally significant in understanding animal behavior. For example, a chimpanzee raised in isolation may develop limited intersubjective capacities compared to one raised in a socially rich environment (Fouts, 1994; Hopper et al., 2016; Thompson González et al., 2021). Similarly, migratory birds may exhibit heightened bodily experiences during navigation, adapting to the demands of long-distance flight and environmental variability (Kishkinev, 2015). Even octopuses exhibit dynamic changes in their situated/extended selves, using environmental tools in creative ways to solve immediate challenges (Amodio, 2019; Finn et al., 2009; Mather, 1994).

## 4 Implications and future directions

We propose adopting a multidimensional perspective for studying the self in animals, emphasizing the graded nature of manifestations within each dimension and the non-hierarchical organization across dimensions proposed by PTS. By accommodating both inter- and intra-species variability, the PTS framework enables researchers to explore the self across a wide range of organisms without imposing anthropocentric biases. This is a feature closely tied to the non-hierarchical character of the self-pattern. No one factor or process in the self-pattern is in principle casually primary or foundational, even if empirically one or more factors, in different cases, can take precedence or can have different weights. PTS provides a lens to explore the multifaceted nature of the self as it manifests across varying ecological and evolutionary contexts, offering an innovative understanding which may contribute to the profound ethical implications already reported about animal rights and welfare (Browning, 2022; Glock, 2019; Godfrey-Smith, 2020; Passmore, 1975). In highlighting these key features, we aim to show how the PTS manages to account for the diverse ways in which environmental pressures, social structures, and cognitive capacities influence the development and expression of the self.

Future empirical investigations may address challenges in operationalizing certain dimensions of the PTS for animal research. Nonetheless, adopting this perspective could drive and stimulate the development of innovative methodologies to explore the diverse aspects proposed by the PTS. By integrating insights from comparative psychology, ethology, and neuroscience (Gosztolai and Ramdya, 2022; Lei, 2023; Michel and Moore, 1995; Proust, 2019), researchers could devise new methods to investigate how these dimensions evolve in animals over time. Higher-order self-continuity can be studied via longitudinal behavioral tracking (GPS-based movement, accelerometry, ethograms) and memory/planning tasks with neuroimaging (e.g., Hanks and Summerfield, 2017; Murray and Rudebeck, 2018). Video monitoring and neural recordings may detect neurophysiological synchrony linked to affiliative behaviors and cooperation (Foster et al., 2011; Hansmeyer et al., 2023). Additionally, cross-species comparisons might uncover adaptive patterns that are intricately linked to behavioral and environmental pressures. Research should adopt hybrid paradigms integrating observational, behavioral, physiological, and neural measures. Neural synchrony and behavioral tracking can reveal how sensorimotor and proprioceptive processes contribute to self-representation (Freiwald, 2020; Kingsbury et al., 2019). Electrophysiological measures with motion-tracking may identify cortical and subcortical activity linked to self-processing, even in species lacking explicit self-recognition (Kim et al., 2017). Self-agency detection paradigms help clarify how organisms distinguish self-generated vs. external actions (Cooper et al., 1999; Szabo and Ringler, 2023), while autonomic responses (HRV, ECG, oxytocin, cortisol) paired with cognitive and social paradigms can assess affective and social engagement (McCall and Singer, 2012; Ziegler and Crockford, 2017). Finally, machine-learning-based investigations offers a scalable approach for detecting species-specific self-pattern, tracking measures of different or specific self-aspects on multiple dimensions (Besson et al., 2022; Maekawa et al., 2020).

These studies may explore the role of language both intra and across species. Many theories posit that self-aspects like narrative and reflective are closely tied to linguistic abilities (e.g., Hirsh and Peterson, 2009; Kerby, 1991). The PTS, accommodating both linguistic and non-linguistic features, predicts that animals may exhibit structured behaviors that may serve as alternative, non-verbal forms of specific self-related aspects. For example, social learning in dolphins (Janik and Sayigh, 2013), tool use in primates (Whiten et al., 1999), and culturally transmitted behaviors in whales (Rendell and Whitehead, 2001) suggest that self-related cognitive and social structures can emerge without linguistic mediation. However, research on animal vocal language suggested that species like bottlenose dolphins, parrots, and marmosets use stable vocal labels, enabling individual recognition and social differentiation (Balsby et al., 2012; Bruck et al., 2022; Seyfarth et al., 2010; Oren et al., 2024). Future studies could explore how these vocal labels are linked to self-aspect and relate to self-identity differentiation (e.g., Agamaite et al., 2015; Pardo et al., 2024; Jaakkola, 2025).

Finally, we would like to briefly highlight that the non-hierarchical and multidimensional nature of PTS makes it relevant for future inquiries on the self not only in non-human biological systems (like animals) but also in non-biological systems, such as generative artificial intelligence (AI), particularly Large Language Models (LLMs). LLMs are disembodied transformer-based networks trained on vast text corpora to predict tokens and generate coherent, human-like dialog when embedded in systems like chatbots (Orrù et al., 2023; Shanahan, 2024). Their ability to simulate human communication is so compelling that recent studies have shown that people often ascribe a genuine form of self-awareness to LLMs (e.g., Colombatto and Fleming, 2024; Floridi and Chiriatti, 2020; Guingrich and Graziano, 2024; LeDoux et al., 2023; Shanahan et al., 2023). Although LLMs lack a biological body and likely self-awareness (e.g., Aru et al., 2023), future research could investigate whether PTS can be functionally applied to human-LLM interactions to enhance our understanding the dynamics of self-attribution to AI.

## Data Availability

The original contributions presented in the study are included in the article/supplementary material, further inquiries can be directed to the corresponding author.
